# Supplementary material for: Calcium Kidney Stones are Associated with Increased Risk of Carotid Atherosclerosis: The Link between Urinary Stone Risks, Carotid Intima-Media Thickness, and Oxidative Stress Markers
Source: J Clin Med. 2020 Mar 8;9(3):729. doi: 10.3390/jcm9030729 (PMC7141231; doi:10.3390/jcm9030729)
Supplement: Supplementary file 1 [file jcm-09-00729-s001.pdf]

Supplementary Materials: Calcium Kidney Stones are Associated with Increased Risk of Carotid Atherosclerosis: the Link between Urinary Stone Risks, Carotid Intima-Media Thickness, and Oxidative Stress Markers

Ho Shiang Huang, Pao Chi Liao and Chan Jung Liu *

Table 1. Stone composition in patients with kidney stone group.

Variables	Kidney Stone Group (*n* = 114)
Mean ± SD% stone composition	
CaOx	53.52 ± 39.78
CaP	28.79 ± 33.66
Ammonium magnesium	9.56 ± 29.40
UA	4.47 ± 20.57
Sodium urate	2.07 ± 13.40
No. containing any composition (%)	
CaOx	87 (76.3)
CaP	91 (79.8)
Ammonium magnesium	11 (9.6)
UA	6 (5.3)
Sodium urate	3 (2.6)
No. pure (%)	30 (26.3)
CaOx	6 (20.0)
CaP	7 (23.3)
Ammonium magnesium	10 (33.3)
UA	5 (16.7)
Sodium urate	2 (6.7)
No. mixed (%)	84 (73.7)
No. greater than 70% mixed (%)	95 (83.3)
CaOx	55 (57.9)
CaP	22 (23.2)
Ammonium magnesium	11 (11.6)
UA	5 (5.3)
Sodium urate	2 (2.1)

SD: Standard deviation; No.: Number; CaOx: Calcium oxalate; CaP: Calcium phosphate; UA: Uric acid.
